# Supplementary material for: Structure–behaviour correlations between two genetically closely related snail species
Source: R Soc Open Sci. 2020 Jan 22;7(1):191471. doi: 10.1098/rsos.191471 (PMC7029891; doi:10.1098/rsos.191471)

**SUPPORTING INFORMATION to:**

**Structure-behaviour correlations between two genetically closely related snail species**

H. Le Ferrand^1,*^, Y. Morii^2,3^

^1^ School of Mechanical and Aerospace Engineering, School of Materials Science and Engineering, Nanyang Technological University, Singapore

^2^ Phenix Group, School of Agriculture and Environment, Massey University, Private Bag 11-222, Palmerston North 4410, New Zealand

^3^ Department of Forest Science, Research Faculty of Agriculture, Hokkaido University, Sapporo, Hokkaido 0608589, Japan

^*^ To whom correspondence should be addressed : hortense@ntu.edu.sg

**
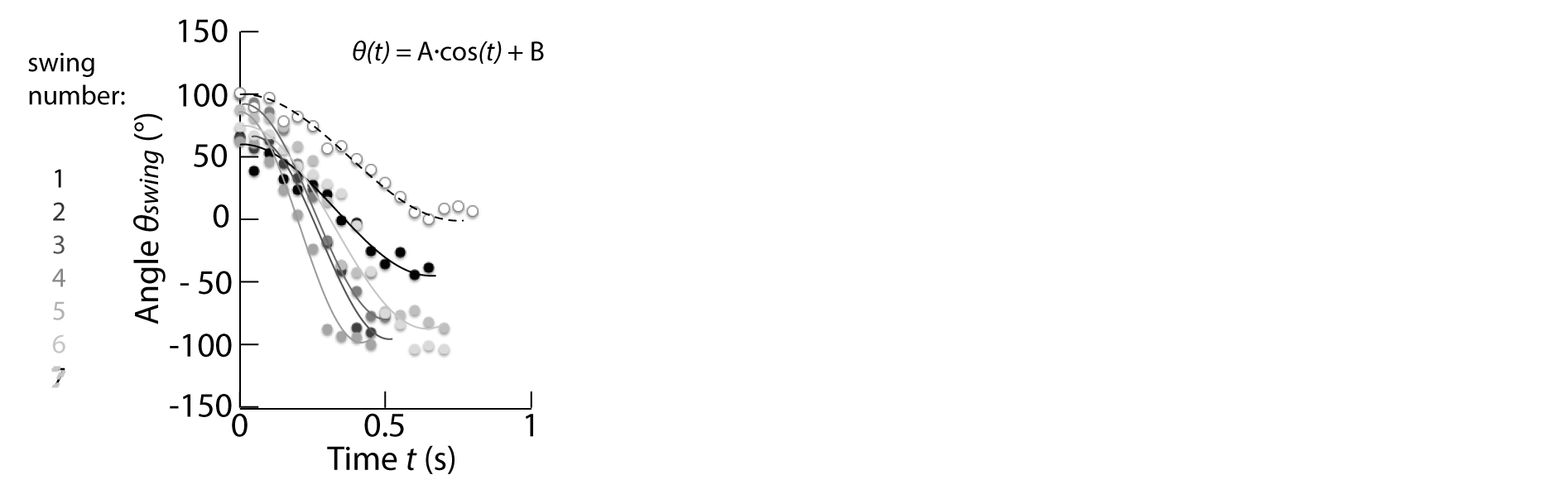
**

**Figure S1.** Angle of the snail shell with respect to the snail body (angle *θ_swing_*) as a function of the time *t* and for 7 consecutive swings, for *K. gainesi*.

**
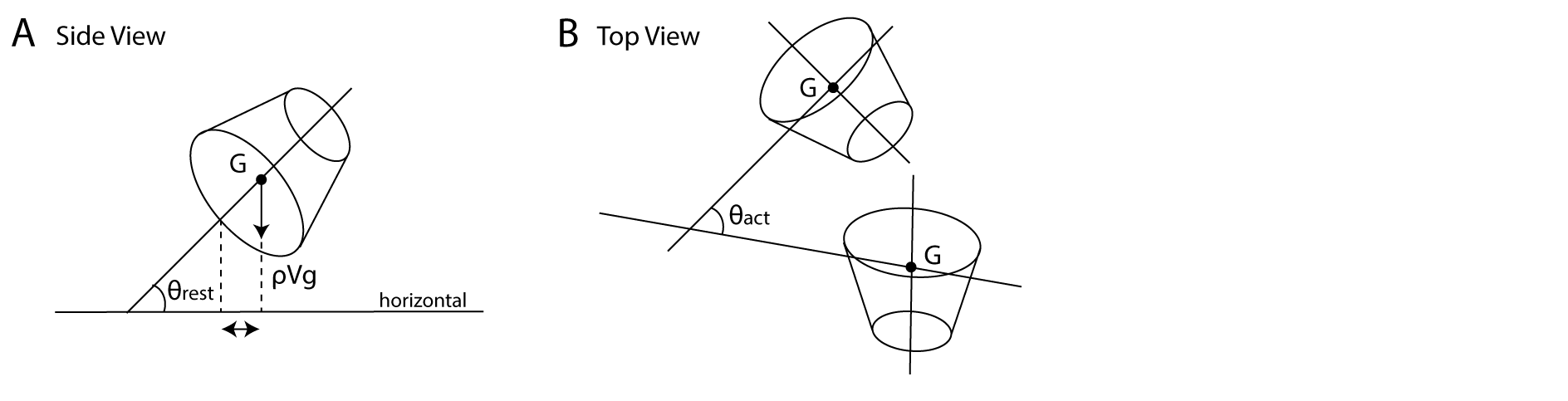
**

**Figure S2.** Schematics for the determination of the shell’s moment when the snail is at rest on a horizontal surface (**A**) and when the snail is swinging its shell (**B**).

**
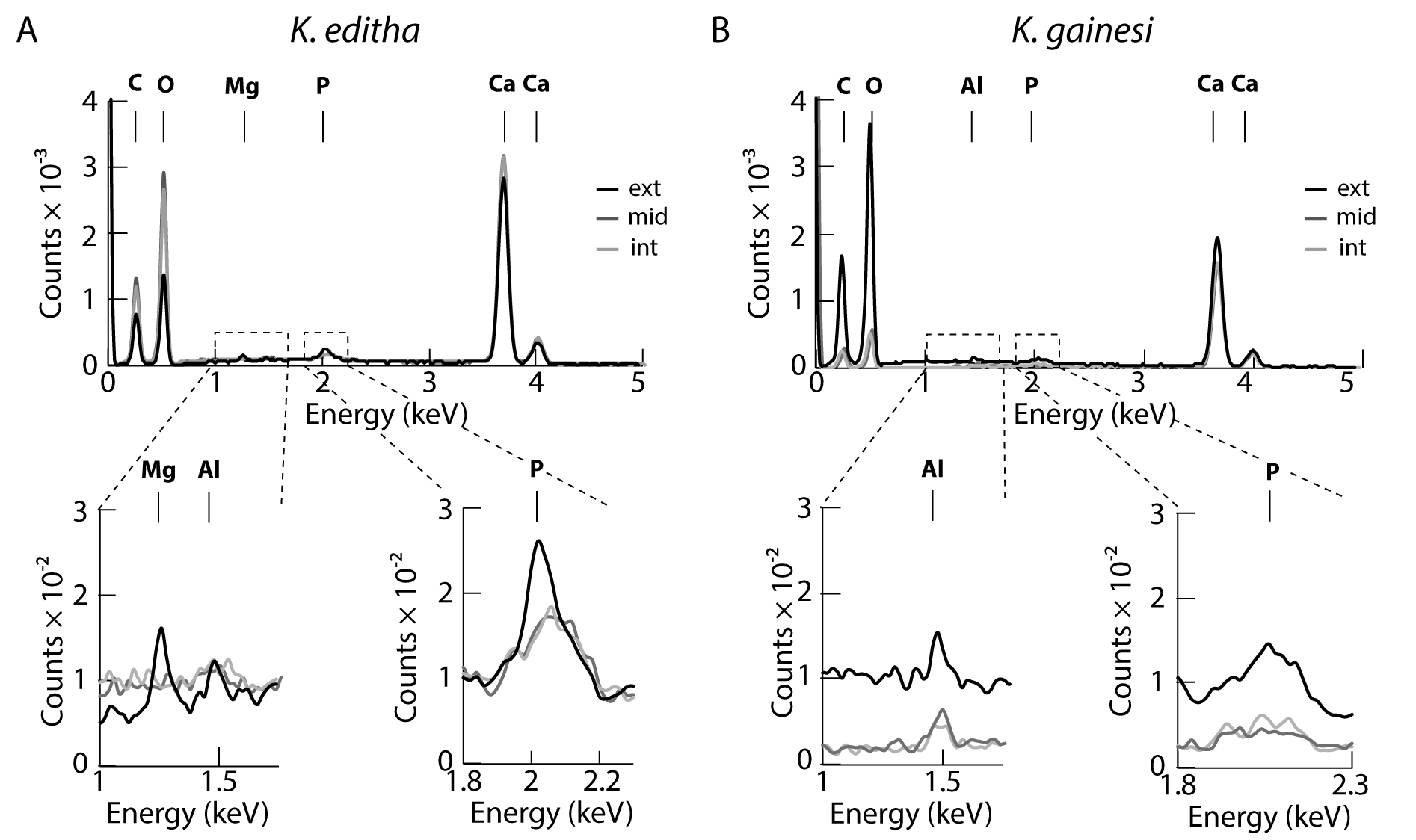
**

**Figure S3.** Energy dispersive X-Ray spectra up to 5 keV obtained for *K. editha* (**A**) and *K. gainesi* (**B**), respectively.

**Table S1.** Collection details of the snails.


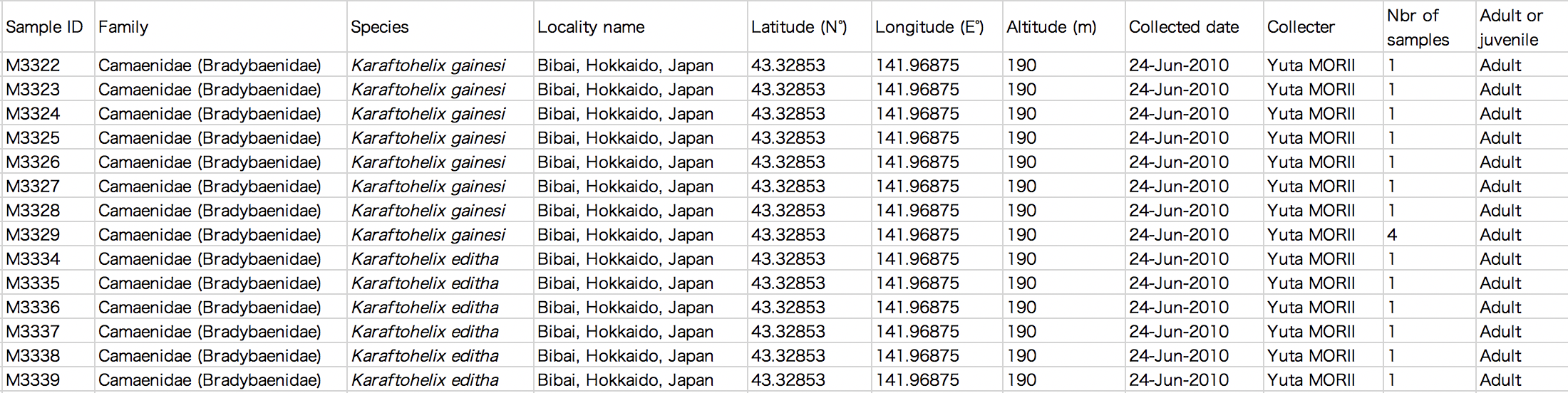

Supplement: Figures S1 - S3 [file rsos191471supp1.docx]
